# Improved reduced representation bisulfite sequencing for epigenomic profiling of clinical samples

**DOI:** 10.1186/1480-9222-16-1

**Published:** 2014-01-09

**Authors:** Yew Kok Lee, Shengnan Jin, Shiwei Duan, Yen Ching Lim, Desmond PY Ng, Xueqin Michelle Lin, George SH Yeo, Chunming Ding

**Affiliations:** 1 Singapore Institute for Clinical Sciences, Agency for Science, Technology and Research (A*STAR), 117609, Singapore, Singapore; 2 KK Women’s and Children’s Hospital, 229899, Singapore, Singapore

**Keywords:** DNA methylation, Epigenetics, Bisulfite sequencing, Clinical sequencing

## Abstract

**Background:**

DNA methylation plays crucial roles in epigenetic gene regulation in normal development and disease pathogenesis. Efficient and accurate quantification of DNA methylation at single base resolution can greatly advance the knowledge of disease mechanisms and be used to identify potential biomarkers. We developed an improved pipeline based on reduced representation bisulfite sequencing (RRBS) for cost-effective genome-wide quantification of DNA methylation at single base resolution. A selection of two restriction enzymes (Taq^α^I and MspI) enables a more unbiased coverage of genomic regions of different CpG densities. We further developed a highly automated software package to analyze bisulfite sequencing results from the Solexa GAIIx system.

**Results:**

With two sequencing lanes, we were able to quantify ~1.8 million individual CpG sites at a minimum sequencing depth of 10. Overall, about 76.7% of CpG islands, 54.9% of CpG island shores and 52.2% of core promoters in the human genome were covered with at least 3 CpG sites per region.

**Conclusions:**

With this new pipeline, it is now possible to perform whole-genome DNA methylation analysis at single base resolution for a large number of samples for understanding how DNA methylation and its changes are involved in development, differentiation, and disease pathogenesis.

## Background

Epigenetics is the study of heritable changes in gene expression without altering the DNA sequence. DNA methylation, by the addition of a methyl group to the carbon-5 position of the cytosine residues, is one important epigenetic regulatory mechanism [1]. In mammalian development, genome-wide *de novo* methylation occurs during germ cell development and implantation, while genome-wide demethylation occurs during primordial germ cell development and shortly after fertilization [2]. DNA methylation and demethylation also occur during cell differentiation and reprogramming [3]. Abnormal DNA methylation levels, either hypermethylation or hypomethylation in specific genes, are also frequently observed in pathologic states, particularly in cancer [4].

Accurate quantification of DNA methylation is essential to decipher mechanisms and pathways regulated epigenetically in development and pathogenesis. Many techniques have been developed for the detection and quantification of DNA methylation [reviewed by [5,6]].

The combination of bisulfite conversion and high-throughput sequencing (Bis-Seq) offers the most quantitative method for DNA methylation analysis at single base resolution. Unmethylated cytosine is converted to uracil by sodium bisulfite treatment while methylated cytosine remains unchanged [7]. Genomic DNA after bisulfite conversion is amplified by PCR and then sequenced at high depth, yielding quantitative measurements of individual cytosine methylation.

While genome-wide Bis-Seq was achieved, published studies typically only analyzed a few samples from cultured cell lines [8-10]. When it is required to analyze tens or hundreds of clinical samples, reduced representation bisulfite sequencing (RRBS) may be the method of choice [11]. The RRBS method makes use of restriction enzyme digestion to selectively analyze genomic regions enriched for CpG sites in a methylation-independent manner, thus achieving a high coverage of CpG rich regions while greatly reducing sequencing read requirement. The current RRBS protocol uses one single enzyme MspI targeting 5′-CCGG-3′ for DNA fragment selection, which results in selectively covering CpG rich regions [12]. However, coverage for non CpG rich regions is generally poor. Recent epigenomic data suggest that CpG poor regions distal to core promoters perform important regulatory functions [8]. Thus, an improvement over the current RRBS protocol to cover CpG poor regions is essential.

In this paper, we performed a comprehensive *in silico* analysis of restriction enzyme digestion of the human genome. A combination of two enzymes, Taq^α^I and MspI, yielded the most desirable coverage of the CpG sites in both CpG rich and CpG not-so-rich regions. We also describe how Bis-Seq data are analyzed. We believe a detailed description of the entire improved RRBS pipeline would greatly facilitate epigenomic studies requiring the analysis of large numbers of samples.

## Results

We made key improvements in genome-wide DNA methylation analysis by RRBS, in both the experiment steps and the data analysis pipeline. Firstly, through extensive *in silico* analysis we chose to digest the human genomic DNA with a combination of two enzymes, MspI and Taq^α^I, which allowed us to cover both CpG islands (CGIs) as well as genomic regions outside CGIs. Secondly, we removed a size range between 198 to 206 bp (with the adaptor) that are repetitive sequence rich. Lastly, we developed a highly automated bioinformatics pipeline with a detailed step-by-step explanation of both quality control and data analysis.

We used the above pipeline for the analysis of a large number of clinical samples such as human placenta, umbilical cord and leukocytes. As an example, we provide data generated from one human buffy coat DNA.

### Selection of restriction enzymes and DNA fragment size range

**Figure 1:**
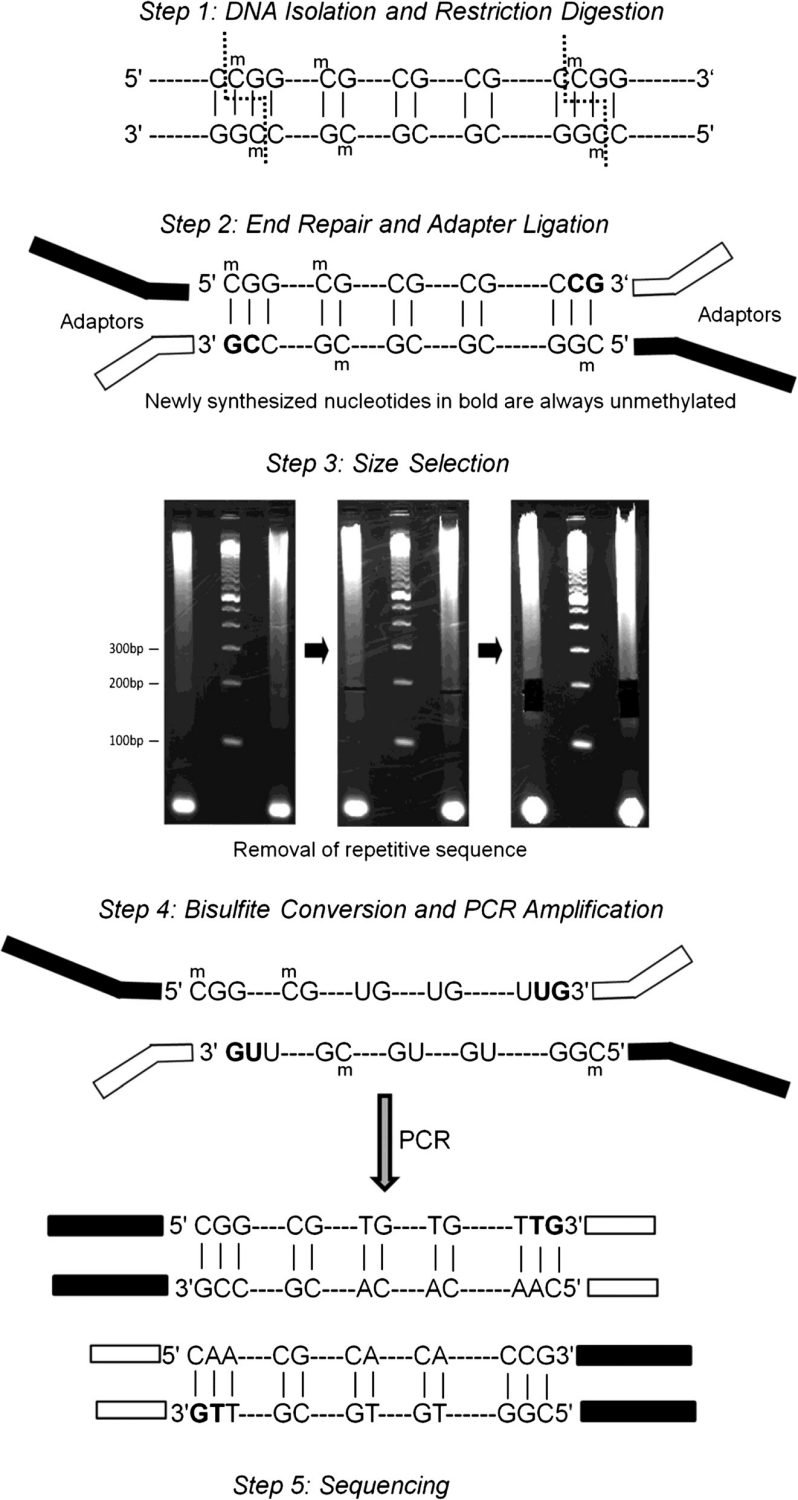
**Key laboratory steps in RRBS.** The isolated DNA from the samples are digested by two restriction enzymes (Taq^α^I and MspI). The fragments are then end-repaired and ligated with adapters. The ligated DNA are size-selected and repetitive sequences are removed. PCR amplification is performed after bisulfite conversion. Illumina GAIIx system is used for high-throughput sequencing. Newly added bases (marked in bold in step 2) are always unmethylated and thus discarded in the downstream analysis for CpG methylation.

We systematically analyzed 289 motifs recognized by restriction enzymes with *in silico* digestion of the human genome (GRCh37/hg19, Feb. 2009 Assembly). Further analyses were carried out with the following considerations: 1) CpG methylation sensitivity and commercial availability of the restriction enzymes; 2) genome-wide coverage of the different genomic regions [promoters (defined as −1000 bp to +500 relative to a transcription start site), CGIs, CpG island shores (CGSs), gene bodies, transcription termination regions (TTRs, defined as −500 bp to +500 bp relative to a transcription termination site)] in the fragments generated by enzyme digestions; 3) size distribution of the fragments; and 4) single enzyme digestion and double enzyme digestions. In the end, MspI and Taq^α^I double enzyme digestion was chosen for the RRBS protocol. A total of 3,810,058 fragments from the human genome are generated by double digestion of the two enzymes, among which 450,689 fragments are between 80 to 160 bp. Within the range of 80 to 160 bp, there is an enrichment of repetitive sequences between 128 to 136 bp, as predicted by *in silico* digestion and validated by a clear DNA band at this size range by gel electrophoresis (around 200 bp with the adaptors) (Figure 1). We removed this DNA band in the lab protocol.

We compared the *in silico* digested DNA fragments (80–160 bp) by MspI and Taq^α^I double digestion and MspI single digestion (single digestion is from the original RRBS protocol). With double digestion, we observed (Table 1) substantial improvements in CpGs in non-CGI regions (41.8% increase), as well as modest improvements in total CpGs in CGIs (7.4% increase), CGIs (6.3% increase) and promoters (12.7% increase).

**Table 1:** **Comparison of *in silico *** **digestions (double vs. single digestion)**

	**Covered by MspI single digestion**	**Covered by MspI and Taq**^**α**^**I double digestion**	**Percentage of increase (double vs. single digestion)**
CpGs in CGI regions	1,098,462	1,180,058	7.4%
CpGs in non-CGI regions	1,919,174	2,720,858	41.8%
CGIs*	20,227	21,511	6.3%
Promoters* (−1000 to +500 bp)	24,520	27,633	12.7%

*by at least 3 CpGs covered in the genomic region.

### Quality control for sequencing results

Our pipeline assesses DNA library and sequencing result quality in a number of different aspects.

#### Quality of sequencing reads

Each flow cell used for DNA sequencing contains eight lanes. Each lane contains 120 tiles. We quantified the number of total reads and the number of good reads (passing a cut-off score of 30) for each tile (Additional file 1: Figure S1). Typically, percentages of good reads were around 70-90%. Additionally, we assessed the percentage of alignment for each library. A typical alignment percentage defined as number of uniquely aligned reads over total pass filtered reads was around 55-65%.

#### Library quality

The preparation protocol for sequencing library by RRBS results in a number of predictable features. Firstly, the first three nucleotides for read 1 of paired-end sequencing should be either CGG/TGG (MspI), or CGA/TGA (Taq^α^I) for the enzymatically generated fragments, while those for read 2 should be CAA. Indeed, these predicted sequencing ends were dominant (97.8% for CGG/TGG/CGA/TGA in read 1 and 91.9% for CAA in read 2) (Additional file 1: Table S1). Secondly, read 1 should map to the positive strand of C2T reference genome, or negative strand of G2A reference genome, while read 2 should be the exact opposite to read 1. This was also confirmed after sequencing alignment (Additional file 1: Table S2). Thirdly, the relative ratio of cytosine over guanine in each read follows a specific pattern. The C/G ratio should be <1 in read 1 and >1 in read 2, as was seen in the sequencing reads (Additional file 1: Table S3). Fourthly, the size distribution of library inserts was analyzed. As expected, the fragment size ranged from 80 to 160 bp, with low abundance shown around 130 bp since we removed the repetitive sequences around this size range (Additional file 1: Figure S2).

#### Bisulfite conversion rate

We estimated the conversion rate using cytosines in non-CpG regions, assuming any unconverted cytosine was due to incomplete conversion. This is likely to over-estimate the non-conversion rate, particularly in samples with cytosine methylation in non-CpG sites, such as in embryonic stem cells [10].

#### Removal of CpGs with potential polymorphisms

As polymorphisms within known CpG sites are likely to cause wrong interpretation of DNA methylation, we implemented a filtering step. A CpG site was discarded if a probable polymorphism was identified. As an example, in one sample we removed 25,135 CpGs out of 1,282,265 CpGs with a minimum sequencing depth of 10.

#### Coverage cutoff

**Figure 2:**
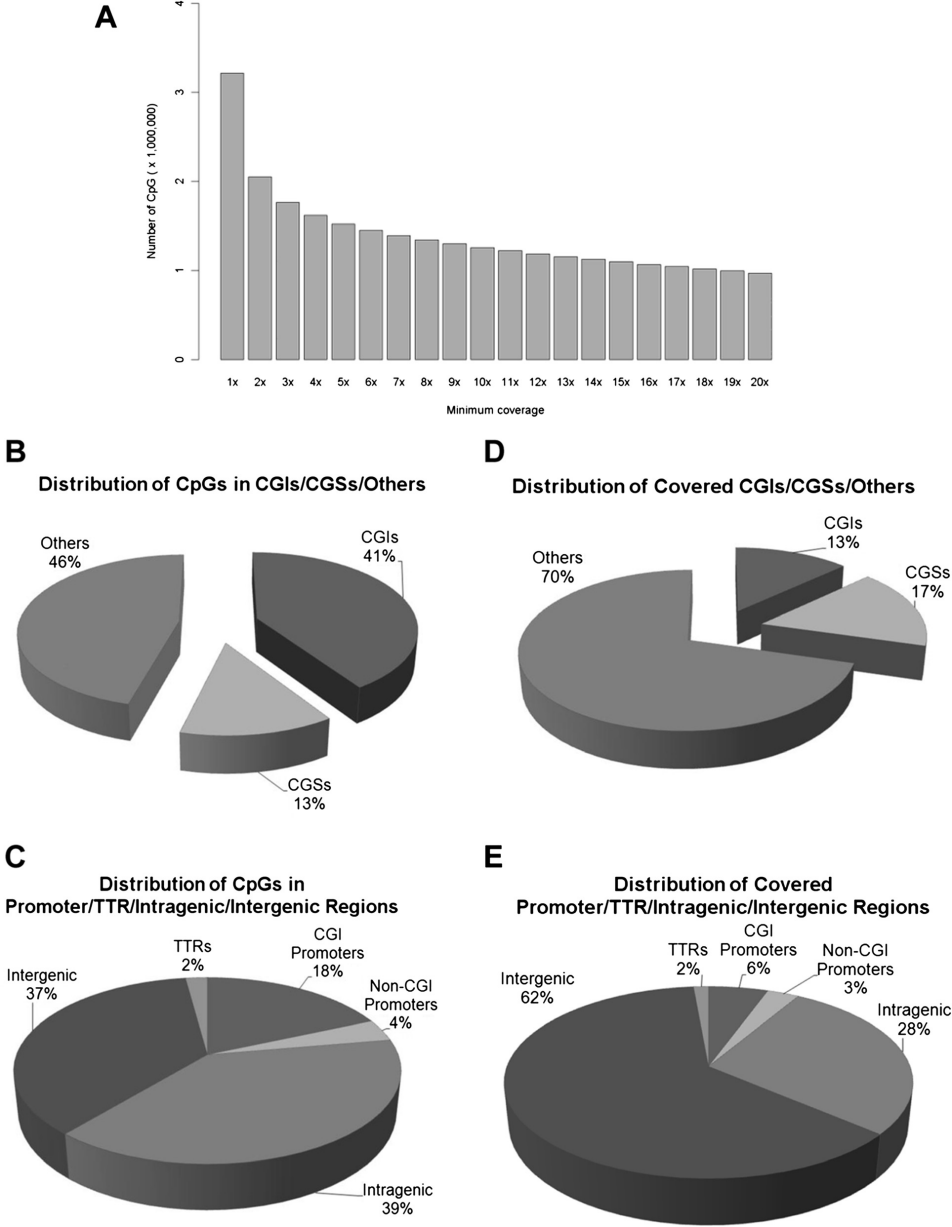
**Coverage of CpGs and genomic regions by RRBS. (A)** An example for number of CpG sites with different minimum sequencing depths; **(B)** Distribution of CpGs in CGIs/CGSs/Others, using a sequencing depth ≥ 10 as the cutoff; **(C)** Distribution of CpGs in Promoter/TTR/Intragenic/Intergenic regions; **(D)** Distribution of genomic regions in CGIs/CGSs/others. **(E)** Distribution of genomic regions in promoter/TTR/Intragenic/Intergenic regions. A genomic region was considered covered if at least three CpGs within the region were sequenced at a depth ≥ 10.

We used a minimum sequencing depth of 10 as the cutoff for inclusion for DNA methylation analysis. However, it is possible that a lower cutoff may be sufficient for many downstream analyses, as suggested by a comparison between RRBS and Illumina Infinium 450 K array [13]. The numbers of CpG sites at different depth were shown in Figure 2A.

### Coverage of CpGs in different genomic regions

Approximately 1.8 million CpG sites with minimum sequencing depth of 10 were included in the analyses. As shown in Figure 2B, 41% of the CpG sites were located within the CGIs, 13% of them were located in CGSs and the remaining CpG sites (46%) were located in CpG poor regions outside of CGIs or CGSs.

As shown in Figure 2C, 18% of the CpG sites were located in the promoter regions overlapping with CGIs while 4% of the CpG sites were located in the promoter regions not overlapping with CGIs. Additionally, 39% of the CpG sites were in intragenic regions, 37% of them were in the intergenic regions and the remaining 2% were in the TTR regions.

Genomic regions with at least three CpGs covered at a minimum sequencing depth of 10 were considered to be covered. With 2 lanes of Solexa sequencing, we achieved 52.2% coverage for core promoters, 76.7% coverage for CGIs and 54.9% coverage for CGSs (Table 2).

**Table 2:** Genome-wide coverage by RRBS

	**Total in human genome**	**Covered by RRBS**	**Percentage**
CpGs	56,434,896	1,837,502	3.3%
CGIs	27,718	21,252	76.7%
CGSs (±2 KB from CGI)	49,300	27,074	54.9%
Promoters (−1000 to +500 bp)	44,399	23,168	52.2%

As shown in Figure 2D, 13% of the covered regions overlapped with CGIs, 17% of them overlapped with CGSs and the remaining regions (70%) were located outside of CGIs or CGSs.

As shown in Figure 2E, 62% of covered regions were in the intergenic regions. Only 6% of the covered regions were in the promoters overlapping with CGIs and 3% were in the promoters not overlapping with any CGI. Twenty eight percent of the regions were found in the intragenic regions and the remaining 2% were in the TTR regions.

## Discussion

Epigenetics changes such as chromatin assembly, histone modifications and DNA methylations are mediators between gene and environment by regulating genomic structures and gene transcription. Genome-wide DNA methylation profiling of human cohorts (or epigenome wide association studies, EWASs) may complement genetic association studies in identifying genes involved in complex diseases. A number of platforms are available for “genome-wide” DNA methylome analysis. True genome-wide DNA methylome analysis by bisulfite sequencing represents the most comprehensive coverage, yet it is cost prohibitive for analyzing a reasonable number of samples. Two platforms, Illumina 450 K Infinium Array and RRBS, appear to be the immediate choices for EWASs [14]. One potential problem with the 450 K Array and the current RRBS approach is poor coverage for CpG poor regions, which may miss important regulatory regions [8].

Through extensive *in silico* enzyme digestion of the human genome, we selected a combination of two enzymes (Taq^α^I and MspI) for a more unbiased coverage of the human genome. We further removed a DNA fragment size range enriched for repetitive sequences so that more reads are usable for alignment. We provide here a comprehensive description for the entire process of the improved RRBS pipeline so that it can be easily adopted by researchers. We also extensively compared the RRBS and 450 K Array platforms by analyzing seven umbilical cord DNA samples with both methods [13]. The two methods are highly complementary as they cover mostly different CpGs and genomic regions. Thus, it is possible to combine the two methods for a more complete coverage for EWASs.

## Conclusions

With this new pipeline, it is now possible to perform whole-genome DNA methylation analysis at single base resolution for a large number of samples for understanding how DNA methylation and its changes are involved in development, differentiation, and disease pathogenesis.

## Methods

### Experimental protocol

A schematic view for the key steps in the lab protocol is outlined in Figure 1.

#### DNA isolation and restriction enzyme digestion

One to five microgram of high molecular weight (>10 kb) genomic DNA was used for each library preparation. Each DNA sample was subjected to sequential restriction enzyme digestion with MspI and Taq^α^I (New England Biolabs). Briefly, DNA was first incubated with 150 U of MspI in a 80 μL system containing 1× Buffer 4 for 2 hrs at 37°C, and the enzyme was inactivated by heating at 80°C for 20 min. Additional 20 μL solution containing 150 U of Taq^α^I, 1× Buffer 4 and 1 μL of 100× BSA was added to the same reaction tube, and the DNA sample was further incubated at 65°C for 2 hrs. The second enzyme was then inactivated by heating at 80°C for 20 min. The double-digested product was purified with the QIAquick PCR Purification Kit (QIAGEN GmbH, Germany), and all was used for library preparation.

#### End repair and adapter ligation

The DNA fragments with 5′-CG-3′ overhangs generated by the restriction enzyme digestion were end-repaired, 3′-end-adenylated, and adapter-ligated using the ChIP-Seq Sample Preparation Kit (Illumina). Illumina’s RRBS for Methylation Analysis protocol was followed, except that 10 μL of Early Access Methylation Adapter Oligo (Illumina) was used and the ligation was performed for 15 min at 20°C in the adapter-ligation step.

#### Size selection of adapter-ligated fragments

Two different sizes of fragments (150–197 bp and 207–230 bp) were selected by gel electrophoresis with 3% agarose gel, corresponding to DNA fragments of 80 to 160 bp without the adapter. DNA fragments with high abundance of repetitive sequences (between 198 to 206 bp with the adaptor, based on *in silico* analysis) were removed using GeneCatcher Gel Excision Kit (Gel Company), and the remaining fragments between 150 to 230 bp were purified by MinElute Gel Extraction Kit (QIAGEN GmbH, Germany).

#### Bisulfite conversion and amplification

The purified fragments were then subject to bisulfite conversion using the EZ DNA Methylation-Gold Kit (Zymo Research), according to manufacturer’s instructions. The converted DNA was PCR amplified with 1x reaction buffer, an additional 1.5 mM of MgCl_2_, 300 μM of dNTP mix, 500 nM each of PCR primer PE 1.0 and 2.0, and 2.5 U of HotStarTaq DNA polymerase (QIAGEN). The thermocycling condition was 15 min at 94°C for heat activation, and 8–12 cycles of 20 sec at 94°C, 30 sec at 65°C and 30 sec at 72°C, followed by a 5-min final extension at 72°C. The PCR products were purified by gel electrophoresis.

#### Quantification and quality check of DNA libraries and sequencing

The DNA libraries were quantified using Agilent 2100 Bioanalyzer (Agilent Technologies). Typically final libraries contain 200 fmoles of fragments. Paired-end sequencing (2 × 36 bp) was performed on the Illumina Genome Analyzer IIx platform, as per manufacturer’s instructions.

### Data analysis pipeline

#### Reference genome conversion

The human reference genome was converted into two reference genomes corresponding to the forward strand (Watson Strand) and the reverse strand (Crick Strand). The C2T converted reference genome was derived by converting all cytosines to thymines. The G2A converted reference genome was derived by converting all guanines to adenosines.

#### Initial quality control of sequencing reads

The paired-end 36 bp reads were filtered based on their Phred scores [15], using a cutoff of 30 [16] which indicates a base calling error of 0.001.

The quality of reads was examined by plotting the number of total reads and the numbers of reads passing the quality cut-off score from each of the 120 tiles of each sequencing lane (Additional file 1: Figure S1).

Additionally, enzymatically generated DNA fragments should end with specific tri-nucleotides such as CGG/TGG/CGA/TGA/CAA while DNA fragments from random shearing may end with any sequence. Thus sequencing library quality was also assessed by calculating the percentages of reads ending by expected tri-nucleotides.

#### Reads conversions

The design of the assays predicts that read 1 sequence should have a C/G ratio < 1 while read 2 sequence should have a C/G ratio > 1, if bisulfite conversion is complete and there is no non-CpG cytosine methylation. Thus, only paired-end reads that followed the above rules were used.

#### Sequence alignment

**Figure 3:**
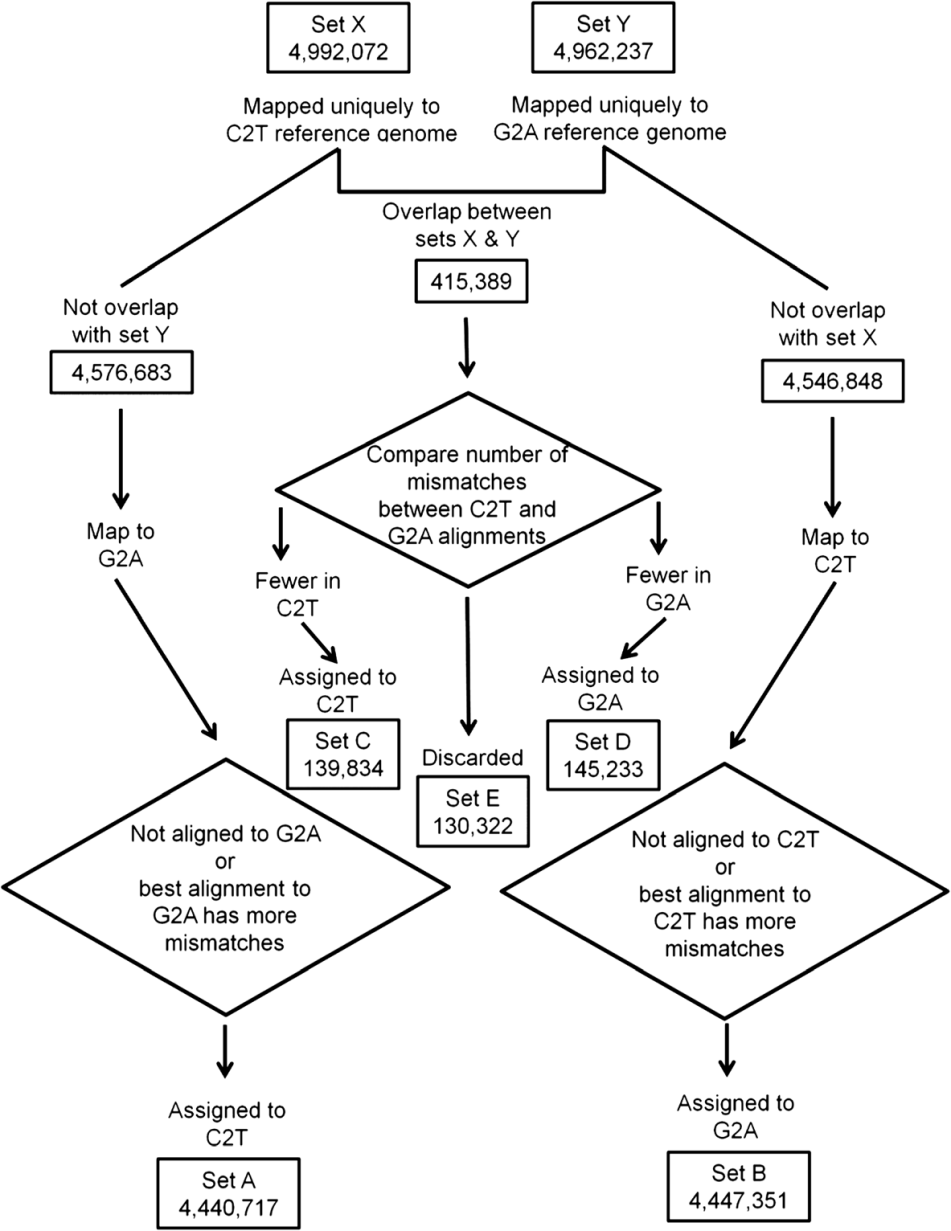
**Sequencing reads alignment to two reference genomes, C2T and G2A reference genomes.** Due to the pointer size limitation by the Bowtie program, reads alignment needs to be performed using the two reference genomes separately. Cross check and comparison are made subsequently to remove reads not uniquely aligned. At the end, sets A and C are read pairs aligned to C2T reference genome, while sets B and D are read pairs aligned to the G2A reference genome.

Bowtie, an ultrafast, memory-efficient short read aligner was used for aligning the sequencing reads [17]. Unfortunately, Bowtie cannot hold a combined index for both C2T and G2A reference genomes due to the fact that it uses a 32-bit pointer and thus it can handle up to a theoretical maximum of 2^32^-1 (slightly more than 4 billions). Thus, two separate Bowtie indexes were created for the C2T and G2A reference genomes, respectively. We thus developed a process for sequencing reads alignment (Figure 3).

All pass-filtered, converted reads were aligned to the C2T and G2A reference genomes separately, using paired-end reads alignments. Reads set X (4,994,072 pairs) were those read pairs that aligned uniquely to the C2T reference genome, while set Y (4,962,237) were those that aligned uniquely to the G2A reference genome. Overlapping read pairs between set X and set Y (415,389) were then compared between the C2T and G2A alignments with regards to the number of mismatched bases, resulting in further separation of sets C (139.834), D (145,233) and E (130,322). The read pairs in set X not overlapping with set Y were then aligned to the G2A reference genome for further comparison, resulting in set A (4,440,717). Similarly, set B was obtained.

At the end, sets A and C were read pairs aligned to C2T reference genome, while sets B and D were read pairs aligned to the G2A reference genome. All other read pairs were discarded since they were not able to align uniquely to the combined C2T and G2A reference genomes.

#### Excluding newly filled-in Cytosines

The newly added cytosines in the “End Repair” step are always unmethylated, regardless of their original methylation status. Such cytosines were excluded in our analysis (Figure 1).

#### Removing of CpGs overlapping with potential polymorphisms

Polymorphisms overlapping with CpGs may introduce abnormalities in the data. In this regard, CpG sites with percentage of dinucleotide ‘XY’ other than ‘CG’ or ‘TG’ greater than 20% were deemed to be probably polymorphic for the sample and were excluded for further analysis.

#### Calculating each CpG sequencing depth and methylation level

Methylation level of each CpG site was calculated as below:

Methylation level for a CpG = Count of Cytosine/ (Count of Cytosine + Count of Thymine)*100%.

Cytosines in non-CpG positions were used to calculated bisulfite conversion rate, assuming any unconverted cytosine was due to incomplete conversion.

The pipeline is available for academic, non-commercial use upon request.

## Abbreviations

RRBS: Reduced representation bisulfite sequencing; Bis-Seq: Bisulfite conversion and high-throughput sequencing; CGIs: CpG islands; CGSs: CpG island shores; TTRs: Transcription termination regions; EWASs: Epigenome wide association studies.

## Competing interests

The authors declare that they have no competing interests.

## Authors’ contributions

YKL, SD and YCL performed the computational analysis. SJ, DPYN and XML performed the experiments. GSHY provided the samples. YKL, SJ and CD wrote the paper. All authors read and approved the final manuscript.

## Supplementary Material

Additional file 1: Figure S1 Assessment of Reads Quality for Each Sequencing Run. Good: read pairs passing the Phred score cutoff of 30. Total: total raw read pairs. **Figure S2**. Distribution of Library Insert Length. **Table S1**. Counts of the first three nucleotides in the sequencing reads. For read 1, the first three nucleotides were expected to be CGG/TGG/CGA/TGA. For read 2, the first three nucleotides were expected to be CAA. **Table S2**. Number of reads aligned to the positive or negative strand of the two converted reference genomes. C2TRef: C2T reference genome; G2ARef: G2A reference genome. **Table S3**. C/G ratios in the sequencing reads. In the first three cases (row 2 to 4), the read pairs showed expected C/G ratios and were thus used for alignment. All others (row 5) were excluded for further analysis. Click here for file
