# Supplementary material for: Improved reduced representation bisulfite sequencing for epigenomic profiling of clinical samples
Source: Biol Proced Online. 2014 Jan 9;16:1. doi: 10.1186/1480-9222-16-1 (PMC3895702; doi:10.1186/1480-9222-16-1)
Supplement: Additional file 1: Figure S1 — Assessment of Reads Quality for Each Sequencing Run. Good: read pairs passing the Phred score cutoff of 30. Total: total raw read pairs. Figure S2. Distribution of Library Insert Length. Table S1. Counts of the first three nucleotides in the sequencing reads. For read 1, the first three nucleotides were expected to be CGG/TGG/CGA/TGA. For read 2, the first three nucleotides were expected to be CAA. Table S2. Number of reads aligned to the positive or negative strand of the two converted reference genomes. C2TRef: C2T reference genome; G2ARef: G2A reference genome. Table S3. C/G ratios in the sequencing reads. In the first three cases (row 2 to 4), the read pairs showed expected C/G ratios and were thus used for alignment. All others (row 5) were excluded for further analysis. [file 1480-9222-16-1-S1.docx]

# Additional file

# Improved reduced representation bisulfite sequencing for epigenomic profiling of clinical samples

### Yew Kok Lee^1^*, Shengnan Jin^1^*, Shiwei Duan^1^, Yen Ching Lim^1^, Desmond P.Y. Ng^1^, Xueqin Michelle Lin^1^, George S.H. Yeo^2^, Chunming Ding^1§^

^1^Singapore Institute for Clinical Sciences, Agency for Science, Technology and Research (A*STAR), 117609, Singapore

^2^KK Women's and Children's Hospital, 229899, Singapore

*These authors contributed equally to this work

^§^Corresponding author

Email address:

CMD: [cmding@gmail.com](mailto:cmding@gmail.com)

Additional file 1: Figure S1. Assessment of Reads Quality For Each Sequencing Run. Good: read pairs passing the Phred score cutoff of 30. Total: total raw read pairs.

Additional file 1: Figure S2. Distribution of Library Insert Length.

For all the reads:

Additional file 1: Table S1. Counts of the first three nucleotides in the sequencing reads. For read 1, the first three nucleotides were expected to be CGG/TGG/CGA/TGA. For read 2, the first three nucleotides were expected to be CAA.


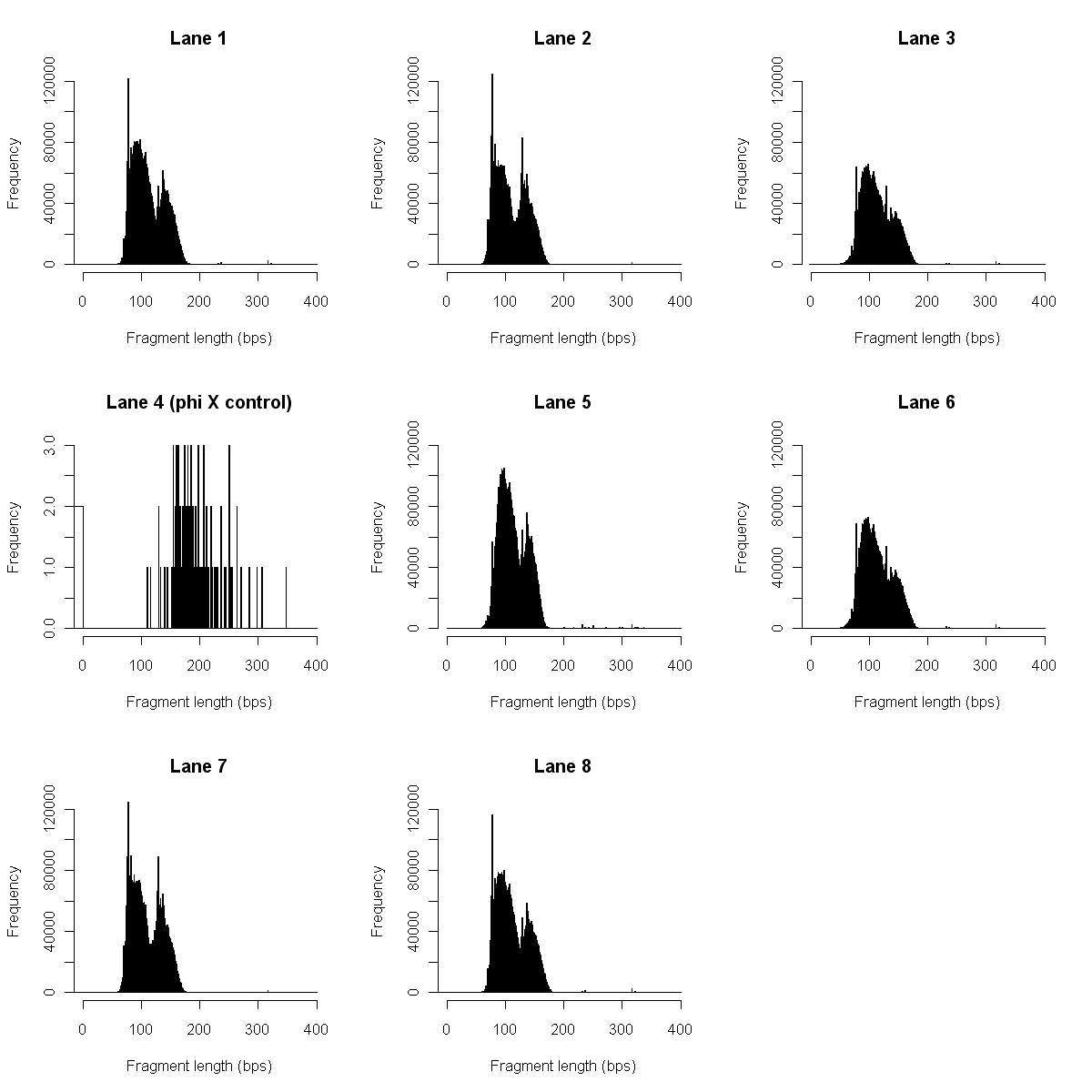


| Lane 1 Read 1 |  | Lane 1 Read 2 |  |
| --- | --- | --- | --- |
|  |  |  |  |
| Sum of All Counts | 10,681,586 | Sum of All Counts | 10,681,586 |
|  |  |  |  |
| Counts | First 3 nucleotides | Counts | First 3 nucleotides |
| 3,966,858 | CGG | 9,813,335 | CAA |
| 3,532,742 | TGG | 490,818 | CGA |
| 1,750,839 | CGA | 68,653 | NAA |
| 1,193,603 | TGA | 61,920 | CAG |
| 28,342 | TTT | 43,173 | CGG |
| 13,775 | GTT | 23,824 | AAA |
| 13,482 | TAG | 18,797 | CCA |
| 11,647 | CAG | 17,442 | TAA |
| 11,367 | TTA | 14,305 | NNN |
| 11,262 | TAT | 12,984 | CTA |
| 10,403 | TAA | 9,803 | CAC |
| 10,205 | TTG | 9,024 | CCC |
| 8,412 | GGT | 7,682 | CCT |
| 8,176 | TGT | 7,323 | CAT |
| 7,699 | AGG | 7,111 | AAC |
| 6,922 | ATT | 6,465 | AAT |
| 6,907 | AAA | 5,544 | ACC |
| 6,852 | GGG | 5,220 | ACA |
| 6,325 | GAT | 4,986 | NNA |
| 6,168 | GGA | 4,804 | ACT |
| 6,001 | GAG | 4,049 | CTT |
| 5,560 | GAA | 3,817 | CTC |
| 5,072 | CAA | 3,610 | TCA |
| 4,912 | AGT | 3,572 | ATA |
| 4,698 | GTA | 3,083 | NGA |
| 4,147 | AAT | 3,074 | TAC |
| 3,645 | GTG | 2,790 | TAT |
| 3,563 | AGA | 2,690 | ATT |
| 3,223 | ATA | 2,630 | TTT |
| 2,819 | AAG | 2,567 | TCC |
| 2,649 | CGT | 2,306 | TCT |
| ... | ... | ... | ... |
| ... | ... | ... | ... |
| ... | ... | ... | ... |
| ... | ... | ... | ... |
| Total Percentage  (CGG + TGG + CGA + TGA) | 97.8% | Total Percentage  (CAA) | 91.9% |

Additional file 1: Table S2. Number of reads aligned to the positive or negative strand of the two converted reference genomes. C2TRef: C2T reference genome; G2ARef: G2A reference genome.

|  | C2TRef | | | | G2ARef | | | |
| --- | --- | --- | --- | --- | --- | --- | --- | --- |
|  | Read 1 | | Read 2 | | Read 1 | | Read 2 | |
| S1 | 2 | - | 3,412,630 | - | 3,406,274 | - | 3 | - |
|  | 3,412,630 | + | 2 | + | 3 | + | 3,406,274 | + |
| S2 | 1 | - | 3,143,620 | - | 3,139,936 | - | 1 | - |
|  | 3,143,620 | + | 1 | + | 1 | + | 3,139,936 | + |
| S3 | 3,283,607 | + | 3,283,607 | - | 3,274,004 | - | 2 | - |
|  |  |  |  |  | 2 | + | 3,274,004 | + |
| S4 | 1 | - | 3,157,175 | - | 3,144,735 | - | 1 | - |
|  | 3,157,175 | + | 1 | + | 1 | + | 3,144,735 | + |
| S6 | 3 | - | 3,945,575 | - | 3,915,666 | - | 5 | - |
|  | 3,945,575 | + | 3 | + | 5 | + | 3,915,666 | + |
| S7 | 3 | - | 3,955,564 | - | 3,931,783 | - | 3,931,783 | + |
|  | 3,955,564 | + | 3 | + |  |  |  |  |
| S8 | 1 | - | 3,810,620 | - | 3,079,045 | - | 4 | - |
|  | 3,810,620 | + | 1 | + | 4 | + | 3,079,045 | + |

Additional file 1: Table S3. C/G ratios in the sequencing reads. In the first three cases (row 2 to 4), the read pairs showed expected C/G ratios and were thus used for alignment. All others (row 5) were excluded for further analysis.

|  | Lane 1 | Lane 2 | Lane 3 | Lane 4 | Lane 6 | Lane 7 | Lane 8 |
| --- | --- | --- | --- | --- | --- | --- | --- |
| Read 1 C/G < 1, Read 2 C/G > 1 | 99.69% | 99.66% | 99.73% | 99.70% | 99.69% | 99.69% | 99.68% |
| Read 1 C/G = 1, Read 2 C/G > 1 | 0.07% | 0.07% | 0.04% | 0.04% | 0.07% | 0.07% | 0.07% |
| Read 1 C/G < 1, Read 2 C/G = 1 | 0.18% | 0.18% | 0.18% | 0.19% | 0.20% | 0.20% | 0.20% |
| All others | 0.07% | 0.08% | 0.05% | 0.06% | 0.05% | 0.05% | 0.06% |
